# SVM-DO: identification of tumor-discriminating mRNA signatures via support vector machines supported by Disease Ontology

**DOI:** 10.55730/1300-0152.2670

**Published:** 2023-12-14

**Authors:** Mustafa Erhan ÖZER, Pemra ÖZBEK SARICA, Kazım Yalçın ARĞA

**Affiliations:** 1 Department of Bioengineering, Faculty of Engineering, Marmara University, İstanbul, Turkiye; 2 Genetic and Metabolic Diseases Research and Investigation Center, Marmara University, İstanbul, Turkiye; 3 Health Biotechnology Joint Research and Application Center of Excellence, İstanbul, Turkiye

**Keywords:** Cancer, cancer diagnosis, support vector machine, Disease Ontology, feature selection, differential gene expression

## Abstract

**Background/aim:**

The complicated nature of tumor formation makes it difficult to identify discriminatory genes. Recently, transcriptome-based supervised classification methods using support vector machines (SVMs) have become popular in this field. However, the inclusion of less significant variables in the construction of classification models can lead to misclassification. To improve model performance, feature selection methods such as enrichment analysis can be used to extract useful variable sets. The detection of genes that can discriminate between normal and tumor samples in the association of cancer and disease remains an area of limited information. We therefore aimed to discover novel and practical sets of discriminatory biomarkers by utilizing the association of cancer and disease.

**Materials and methods:**

In this study, we employed an SVM classification method for differentially expressed genes enriched by Disease Ontology and filtered nondiscriminatory features using Wilk’s lambda criterion prior to classification. Our approach uses the discovery of disease-associated genes as a viable strategy to identify gene sets that discriminate between tumor and normal states. We analyzed the performance of our algorithm using comprehensive RNA-Seq data for adenocarcinoma of the colon, squamous cell carcinoma of the lung, and adenocarcinoma of the lung. The classification performance of the obtained gene sets was analyzed by comparison with different expression datasets and previous studies using the same datasets.

**Results:**

It was found that our algorithm extracts stable small gene sets that provide high accuracy in predicting cancer status. In addition, the gene sets generated by our method perform well in survival analyses, indicating their potential for prognosis.

**Conclusion:**

By combining gene sets for both diagnosis and prognosis, our method can improve clinical applications in cancer research. Our algorithm is available as an R package with a graphical user interface in Bioconductor (https://doi.org/10.18129/B9.bioc.SVMDO) and GitHub (https://github.com/robogeno/SVMDO).

## 1. Introduction

Cancer is a disease caused by abnormal cell proliferation and loss of normal cell regulation. Due to the dynamic nature of the disease, it can be challenging to diagnose and treat it in its early stages for most patients (Student and Fujarewicz, 2012). Fortunately, molecular-based approaches such as transcriptomics offer the opportunity to study the activity of multiple genes and thus determine early physiological information about cancer (Student and Fujarewicz, 2012; Xiong et al., 2021). A transcriptome is the set of transcripts in a particular tissue or cell of an organism that represents the information flow of gene expression. Large amounts of transcriptome data are available for many phenotypes, including cancer (Dong and Chen, 2013). The information obtained from the transcripts contains specific patterns that reveal the occurrence of certain events hidden in the complex biological architecture (Kukurba and Montgomery, 2015; He et al., 2018). To this end, statistical and machine learning (ML) methods have been used to analyze biological datasets. Researchers have utilized transcriptomic profiles in this field to classify cancer and overcome the limited diagnostic capabilities of conventional methods (Hossain et al., 2021). However, the high dimensionality and small sample size of gene expression datasets pose a challenge for classification approaches that rely on traditional statistical methods (Student and Fujarewicz, 2012).

Among ML approaches, the support vector machine (SVM) algorithm is one of the widely used classification methods enabling subtle pattern recognition in complex datasets. The SVM algorithm creates a decision boundary, called a hyperplane, which divides the entire dataset into two classes to predict the labeling of the data points. Support vectors are the data points closest to the hyperplane from both sides. The distance between the two sides of the support vectors is called the margin. When the margin is large, good classification performance is achieved (Huang et al., 2018).

While a linear separation of the data is desirable in classification procedures, in practice nonlinear classification often occurs. To solve this problem, SVM techniques use kernel methods that map linearly inseparable data points into higher dimensional spaces, making them linearly separable (Zhang et al., 2004). The choice of kernel method has a major impact on classification performance. Unfortunately, there is no exact way to determine which kernel method is better at the beginning. The best kernels can only be selected by experimenting with the dataset (Huang et al., 2018). Among the kernel methods, radial basis function (RBF) is a common classification approach. The RBF kernel provides a nonlinear mapping of data points in a higher dimensional space. It is an effective kernel method when there is a nonlinear relationship between class labels and attributes (Apostolidis-Afentoulis, 2015). In addition, the RBF kernel also provides simplified tuning by using only two parameters: gamma (γ), which adjusts the smoothness of the hyperplane by changing its flexibility (Shadeed et al., 2020), and the penalty parameter (C), which adjusts the tolerance to data points shifted from their sides (Yang et al., 2021).

The SVM algorithm also poses challenges. As the size of the training dataset increases, SVM classification becomes more and more memory-intensive. Moreover, SVMs cannot handle large datasets when kernel methods are involved in the classification process (Yue et al., 2003). Therefore, the dimensions of the involved datasets should be adjusted. Moreover, class imbalance is a problem for SVMs, which leads to high misclassification rates of minority classes (Imam et al., 2006). Transcriptome-based supervised classification studies, including those utilizing SVMs, have mainly used microarray datasets. Despite their low costs, the background noise and signal saturation of microarrays are problematic as they compromise the true potential of using transcriptome data for classification (Zhao et al., 2014). Thanks to emerging next-generation sequencing technologies, RNA-Seq datasets could be useful to minimize these noise reduction issues (Hrdlickova et al., 2017).

Supervised classification may not require all features in large datasets, as not all features may contain sufficient information. For instance, RBF-SVM methods are most effective when the number of features is limited (Apostolidis-Afentoulis, 2015). Irrelevant features in high-dimensional datasets can negatively impact the efficiency of the ML process. Knowledge-based approaches such as Gene Ontology (GO) are commonly used in feature selection methods to minimize this problem (Cai et al., 2018; Liang et al., 2018).

In genetic studies, enrichment analysis is not primarily used to harness the power of the clinical aspect of genes with altered expression levels (Shah et al., 2012). In cancer research, this is an important finding due to the frequent cooccurrence of chronic diseases in cancer patients. For example, certain cancers are associated with infections caused by oncoviruses (Kori and Arga, 2020). Diabetes can also contribute to the development of several types of cancer, including colorectal, prostate, and breast cancer (Gallagher and LeRoith, 2015; Tu et al., 2018). It is therefore possible that similar genes are associated with multiple diseases. These associations can be identified through Disease Ontology (DO) enrichment analysis.

DO is an open-source ontology that integrates biomedical data related to human diseases(Yu et al., 2015) . The DO enrichment approach allows users to ask which disease or class of diseases is overrepresented in a particular gene set of interest. For example, in the study by LePendu et al. (2011), tumor-suppressor gene *TP53* was found to be overrepresented in cancer and fibroepithelial neoplasms and was also annotated with specific diseases such as colorectal cancer and Li–Fraumeni syndrome. Compared to other ontologies, DO is used for research on genomic disease associations. In addition, it is an important database for the development of more effective health informatics tools used for diagnostics and prediction of disease phenotypes and drugs (Schriml et al., 2022).

The use of DO alone may not provide a sufficiently informative pattern for classification. Various feature selection methods have been investigated to effectively eliminate irrelevant and redundant features, including combinations of different methods (Liu et al., 2004). One such approach is the use of Wilk’s lambda criterion, which allows moderate filtering of features while gradually combining different features to create a set of multiple features with high discriminative power (Ouardighi et al., 2007).

The detection of genes that can discriminate between normal cells and tumor samples in terms of the association of cancer and disease remains an area of limited information. In this study, we developed an SVM algorithm that filters cancer RNA-Seq datasets based on DO enrichment using the Wilk’s lambda criterion. This method allows us to identify genes that are effective in classifying normal and tumor samples. Our goal was to discover novel and practical discriminatory biomarker candidates by exploiting the association between cancer and disease. To facilitate the use of our approach, we provide a graphical user interface for all users.

## 2. Materials and methods

### 2.1. Algorithm development

The SVM-DO algorithm was implemented in the R programming language (version 4.2.2) using RStudio IDE (Krotov, 2017). The script was developed to work on Windows and Linux operating systems. The selected R packages were obtained from the repositories of CRAN (Hornik, 2012) and Bioconductor (Gentleman et al., 2004).

### 2.2. Differential expression analysis

We employed a diverse set of gene expression datasets that were obtained from various platforms, including microarray and RNA-Seq, and subjected to different preprocessing conditions such as FPKM (Filloux et al., 2014), RPKM (Wagner et al., 2012), MAS5 (Parrish and Spencer, 2004), and RMA (Parrish and Spencer, 2004) (as listed in Table 1).

To evaluate the performance of the algorithm, the Cancer Genome Atlas (TCGA) (Tomczak et al., 2015) and NCBI Gene Expression Omnibus (GEO) (Barrett and Edgar, 2006) databases were accessed, using datasets linked to cancers of the colon (COAD) and lungs (LUSC and LUAD). The normalized RNA-Seq datasets from the TCGA-COAD, TCGA-LUSC, and GSE40419 (GEO-LUAD) datasets were used to obtain gene sets to distinguish tumor samples from normal cells. Other expression datasets were used to evaluate the diagnostic performance of the SVM-DO algorithm.

Statistical analysis of gene expression was performed using the nortest (version 1.0-4) (Gross and Ligges, 2015) and BSDA (version 1.2.1) (Arnholt and Evans, 2021) packages. Conformity to normal distribution was analyzed using the Anderson–Darling test. The Mann-Whitney U test or z-test was applied to test for differential gene expression in the normalized datasets. In addition, the Wald test was used for RNA-Seq count datasets. The determined p-values were adjusted with Benjamin–Hochberg correction. The significance threshold was set as adjusted p < 0.05 and log2FC ≤ –1.5 or log2FC ≥ 1.5 for differential expression. A user-defined input size (n) was used to filter the original gene lists (i.e., up- and downregulated genes) of TCGA-COAD, TCGA-LUSC, and GEO-LUAD prior to feature selection.

### 2.3. DO enrichment analysis

Differentially expressed genes were first screened for significant disease associations using the DOSE package (version 3.24.2) (Yu et al., 2015), which uses human disease annotation maps provided from the HDO.db package (version 0.99.1) (Hu and Yu, 2022) including detailed information on the recent version of the Human Disease Ontology database.

**Figure 1:**
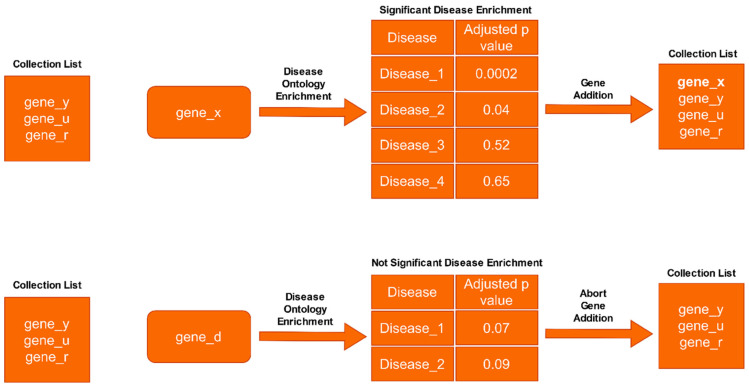
Disease Ontology enrichment analysis in filtering genes.

Disease features and etiological factors are integrated to describe disease complexity (Schriml et al., 2022) and the latest version of the DO database (v2021-08-17) includes 10,862 disease terms and 15 different relationships (disease, phenotype, sequence, etc.). To apply gene set filtration based on significant disease enrichment, adjusted p-values (Benjamin–Hochberg) with a threshold of <0.05 were used (Figure 1).

### 2.4. Gene set trimming and classification

The gene set including features with significant disease enrichment was subjected to additional filtering using the klAR package (version 1.7–2) (Weihs et al., 2005) with the Wilk’s lambda criterion. Using this method, genes were selected based on their individual contributions to the discriminatory model, and each feature was assigned a p-value for its inclusion in the model. This process is optimized by adjusting the “niveau value,” which is the threshold for the p-value of the partial change of the last feature in the model. In our study, an initial level value of 0.1 was used, which was automatically reduced in a gene set trimming loop until it reached the threshold value of ≤0.05. When the current p-values reached the threshold, the process of trimming the gene set was skipped. This process was integrated into a classification model to identify the gene set with the most effective discrimination performance.

Prior to classification, the transcriptome dataset samples were randomly divided into training (80%) and testing (20%) groups using the caTools package (version 1.18.2) (Tuszynski and Khachatryan, 2015). Our classification model used the SVM with a 10-fold cross-validation technique and was created using the e1071 package (version 1.7–13) (Meyer et al., 2023) with RBF. The RBF-SVM parameters gamma (γ, which adjusts the smoothness of the hyperplane) and penalty (C, which adjusts the tolerance) were fine-tuned in the range of (10^−6^, 10^6^) and (10^−5^, 10^5^), respectively. To evaluate the predictive value of the classification model, we created a confusion matrix using the Caret package (version 6.0–94) (Kuhn, 2008) and performed sensitivity analysis by extracting kappa, specificity, and binomial significance tests for the difference between the model accuracy and no information rate (NIR). Thresholds of more than 0.80 were used for kappa and specificity, while thresholds of less than 0.05 were used for the statistical difference between model accuracy and NIR.

### 2.5. Testing for diagnostic performance

To examine model performance, the TCGA datasets and GSE40419 (GEO-LUAD) were used in receiver operating characteristic (ROC) analysis using the precrec package (version 0.14.2) for area under the curve (AUC) scores (Saito and Rehmsmeier, 2017).

In the case that good model performance was achieved, it was decided to assess the reproducibility of the analyses by principal component analysis (PCA). The ggplot2 (version 3.4.2) (Wickham, 2011) and ggpubr (version 0.6.0) (Kassambara, 2020) packages were selected to draw and organize PCA plots. Each gene set was filtered based on the differential expression in the test datasets. In the case that the primary principal components covered at least 80% of the total variance, the metrics for accuracy, specificity, and sensitivity were calculated.

### 2.6. Testing for prognostic performance

The discriminatory gene sets of the TCGA datasets were analyzed for their prognostic performance using survival analyses. For this purpose, subjects were categorized into low- and high-risk groups based on their prognostic index. We performed survival analyses for individual genes using the survival package (version 3.4-0) (Therneau, 2020). The survival signature of each gene was assessed using Kaplan–Meier plots, and a log-rank p-value of <0.05 was used as the cut-off value for statistical significance.

### 2.7. GO and pathway enrichment analysis

Biological mechanisms of gene sets were characterized using GO terms and KEGG pathway enrichment analyses. These analyses were conducted using the Database for Annotation, Visualization, and Integrated Discovery v6.8 (DAVID) online tool (Huang et al., 2009; Sherman et al., 2022). Gene sets were significantly enriched by using a cut-off p-value of <0.05 adjusted by Benjamin–Hochberg correction.

### 2.8. Cancer-related genes in discriminative sets

Enriched disease terms from the initial discriminative gene sets were analyzed using the DOSE package (version 3.24.2) (Yu et al., 2015) to investigate any relatedness to cancer. Benjamin–Hochberg-adjusted p-values with a threshold of <0.05 were used in disease filtering.

## 3. Results

### 3.1. SVM-DO algorithm

**Figure 2:**
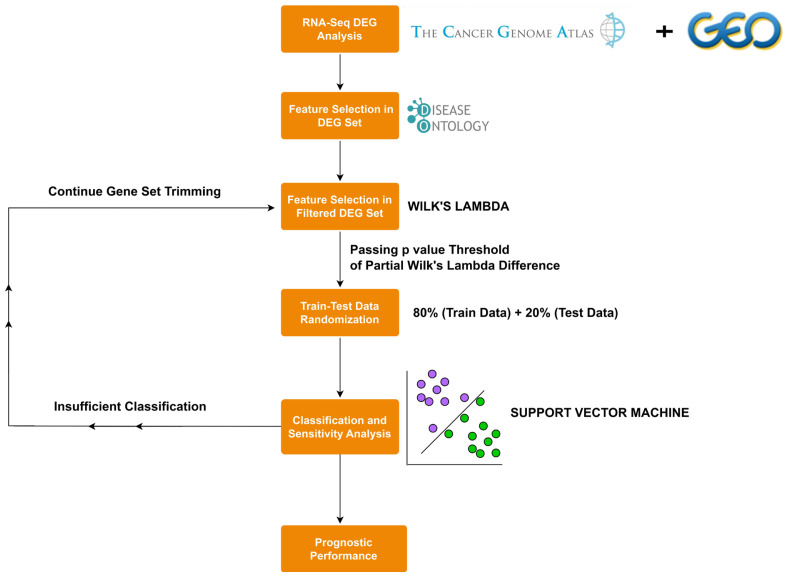
Flow diagram of SVM-DO algorithm.

The algorithm (Figure 2) consists of consecutive steps for differential expression analysis, feature selection, gene set trimming including data randomization and train/test grouping, SVM-based parameter-tuning steps, and prognostic performance analysis. In the first step, differentially expressed genes (DEGs) are extracted from the expression dataset. Secondly, DEGs indicating significant disease enrichment are selected. Following this step, DEGs are applied to Wilk’s lambda criterion-based trimming and SVM-based classification model construction. Based on the classification performance, the final form of the feature set is selected by the algorithm and finally applied to single gene survival analysis to detect genes with prognostic importance.

### 3.2. Effect of input size on simulation duration

**Figure 3:**
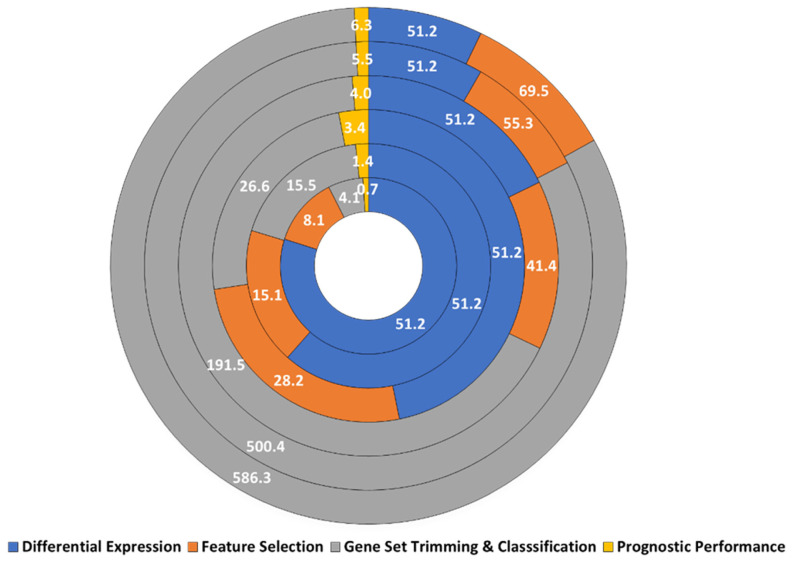
Effect of input size on the execution times (given in seconds) of the simulation steps (inner to outer region: input size of n = 50, 100, 200, 300, 400, and 500).

To evaluate the effectiveness of the acquired discriminative gene set models, we selected input sizes of 50, 100, 200, 300, 400, and 500, which were used after differential expression analysis. The simulations were repeated 10 times and the average durations were calculated. We found that increasing the input size had significant effects on the duration of each step, particularly for gene set trimming and classification (Figure 3).

### 3.3. Evaluation of diagnostic performance

**Figure 4:**
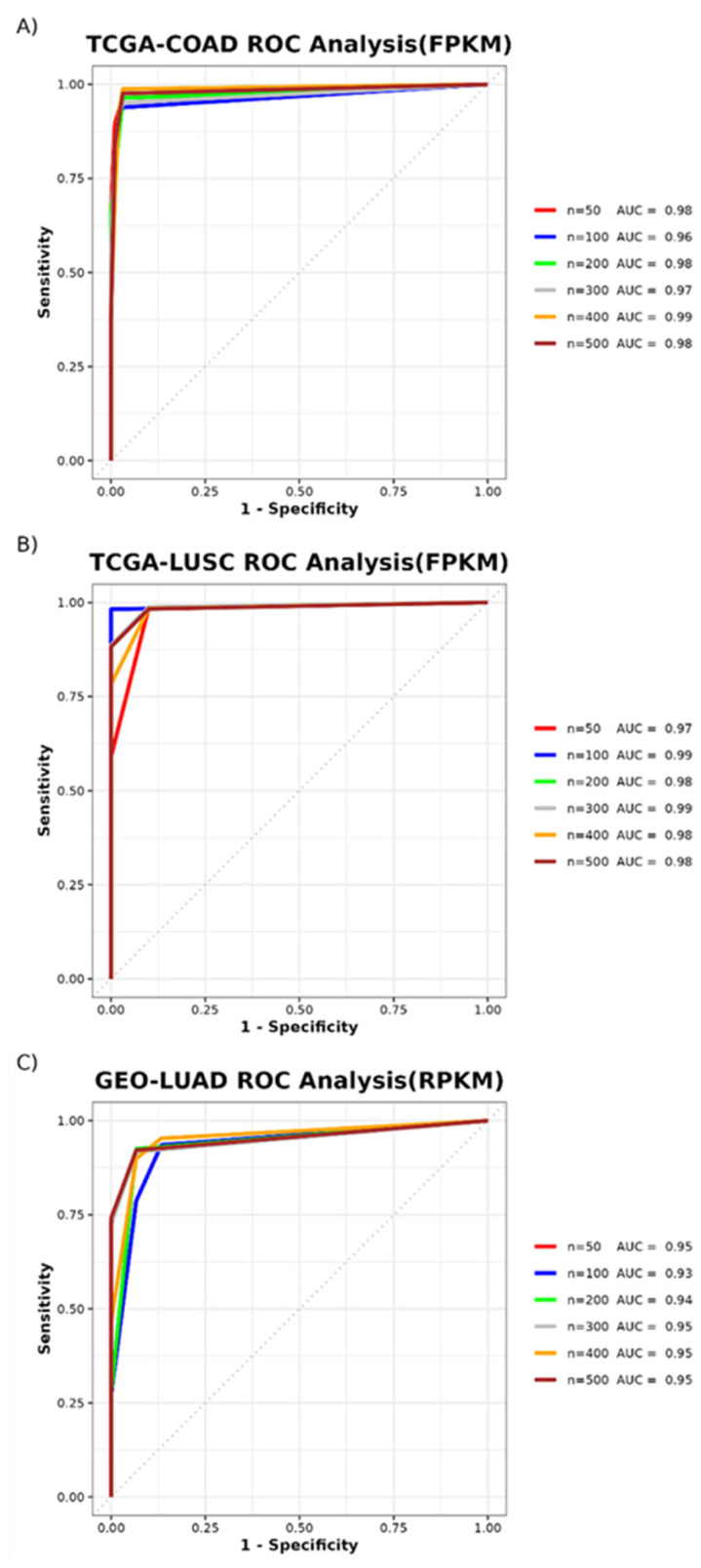
Discriminative performance analysis of algorithm at different input sizes (n) for datasets of TCGA-COAD (A), TCGA-LUSC (B), and GEO-LUAD (C).

The results of the ROC curves illustrated the classification performance of our algorithm between tumor and normal samples using the TCGA-COAD, TCGA-LUSC, and GEO-LUAD datasets. High values (0.93 to 0.99) were observed for the AUCs of each input variable, indicating the high diagnostic accuracy of the algorithm (Figure 4).

Using the PCA results of the datasets (Table 1), sensitivity analysis was performed for each dataset and the averages of the metrics for each input variable were determined (Table 2). The discriminative gene sets obtained from TCGA-COAD, TCGA-LUSC, and GEO-LUAD provided good discrimination between tumor and normal samples in the different expression datasets without significant effects of normalization or platform differences.

### 3.4. Evaluation of prognostic performance

The acquired gene sets from the TCGA datasets showed prognostic effects in individual forms. During the gene set trials, changing the input size affected the number of individual prognostic gene candidates, as provided in Table 3. Despite statistical significance, we observed insufficient prognosis in single gene analyses considering hazard ratios (from 0.6 to 2), as provided in Table 4. However, we were unable to analyze the GSE40419 (GEO-LUAD) dataset due to the lack of survival data and vital statuses of the patients.

### 3.5. Performance comparison with alternative ML methods

**Figure 5:**
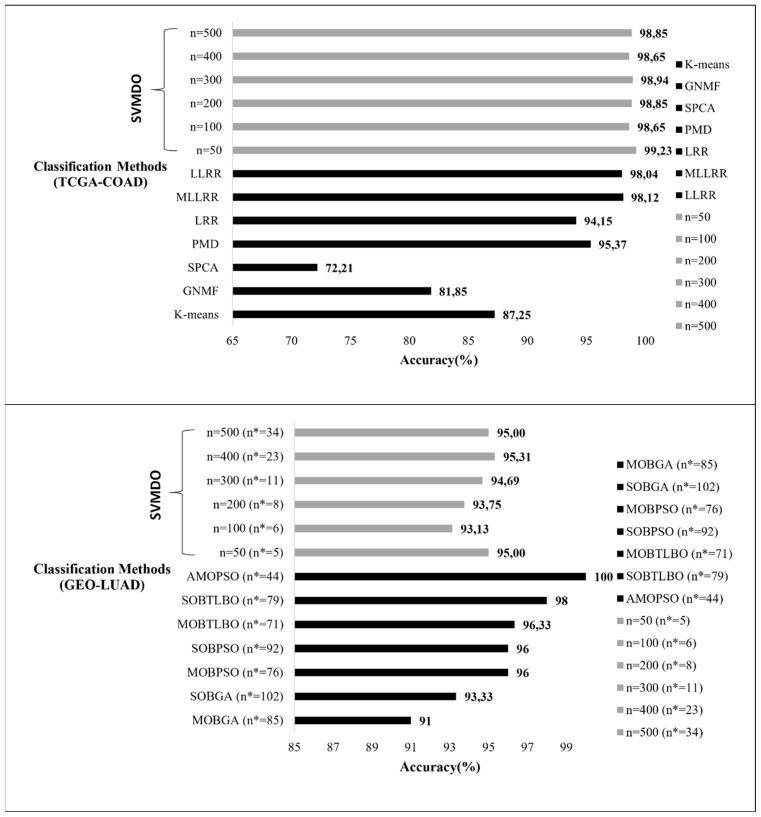
Comparative analyses of TCGA-COAD and GEO-LUAD with discriminative set size (n*).

The performance of the algorithm was compared with existing ML approaches (Table 5) obtained from two previous studies (Shahbeig et al., 2018; Wang et al., 2019). These studies were selected based on precalculated accuracy values for several ML methods using the RNA-Seq datasets of colorectal and lung cancer included in our study. Therefore, the accuracies of the predictions were compared (Figure 5). Our algorithm provided high accuracy values of >98% with gene sets extracted from the colorectal cancer dataset regardless of input size. The overall accuracy of the gene sets was better than that of the previous studies. In contrast, lower performance was observed with the lung cancer dataset (minimum of ~90%, maximum of ~92%) compared to the alternative methods. In the study conducted with the lung cancer dataset, the total number of discriminative gene sets of each ML algorithm was also considered. Our approach resulted in accuracy values of over 90%, with a lower number of genes employed.

### 3.6. GO and pathway enrichment analysis

The gene sets of the TCGA-COAD dataset were mainly enriched in biological functions related to bile secretion and sodium transport, while the KEGG pathway analysis showed enrichment in terms of bile secretion, proximal tubule bicarbonate reclamation, pancreatic secretion, and nitrogen metabolism. On the other hand, the gene sets of the TCGA-LUSC dataset were enriched in biological functions related to the transport of oxygen, carbon dioxide, and nitric oxide; the catabolism of hydrogen peroxide and glutathione; and the leukotriene D4 biosynthesis process. In addition, the malaria pathway was enriched in the KEGG pathway analysis. In contrast, in the GEO-LUAD dataset, there was only one biological function related to neuron projection development. Discriminatory gene sets associated with multiple cancer types were observed (see Supplementary Table S1 for details).

## 4. Discussion

The research field of collecting information on gene–disease associations is constantly evolving. Although techniques utilizing deep learning have shown promising results in detecting such associations, they often do not take into account the multifunctional effects of genes associated with multiple diseases (Chen et al., 2021). Rather than developing complex new techniques, it may be useful to predict the classification ability of a feature set extracted from an existing gene–disease association repository. The method proposed in this study can identify novel disease-related genes while also considering their multifunctional properties. By integrating DO enrichment analysis into our algorithm, we were able to discover various gene–disease relationships.

There are also different methods for integrating disease associations. To understand the differences, we compared SVM-DO with two similar ML-based algorithms, maTE (Yousef et al., 2019) and GediNET (Qumsiyeh et al., 2022), which also use disease associations.

The maTE algorithm was developed to find the best discriminative miRNA set that regulates the target genes and can explain the difference between groups (e.g., cancer vs. control). SVM-DO was developed to find disease-related gene sets that can be used to discriminate between cancer and normal sample groups, but miRNA candidates with distinguishing features could also be obtained by using expression dataset features. Among our results, miR-139 was observed in both discriminative gene lists of the colorectal and lung cancer datasets. miR-139-5p is known as a potential biomarker in the development of several human cancers (Huang et al., 2017) and has been observed to target insulin-like growth factor receptor type I, leading to the inhibition of invasion, metastasis, and cell proliferation in both colorectal cancer and non-small-cell lung cancer (NSCLC) (Shen et al., 2012; Xu et al., 2015). In the maTE algorithm, the involvement of miRNAs in diseases is recognized by an ML approach. In our study, genes with disease associations were selected by DO enrichment analysis.

The GediNET algorithm determines which diseases in a given expression dataset are significantly associated with the major disease of interest. In a sense, our algorithm tries to find discriminative features by associating different diseases with cancer by focusing on disease-related genes. The gene–disease associations analyzed in the study of Qumsiyeh et al. (2022) mainly involved major specific diseases. We wanted to follow a similar approach at the beginning of the development of our algorithm. However, instead of finding genes related to chronic diseases, the genes with the most enriched diseases consisted mainly of annotations related to cancer (carcinomas, tumors, neoplasms, etc.). We wanted to focus on extracting discriminatory features from the gene groups associated with chronic diseases. Therefore, we selected any gene that showed significant enrichment with chronic diseases according to DEG analysis. Measured by the total size of DEGs, this could be a computationally intensive approach. The introduction of a secondary feature selection method reduced the computational burden of the classification process.

When multiple feature detection methods are combined, the results are often unstable, as noted previously (Saha et al., 2021). For example, extracting random gene sets from the same high-dimensional gene expression dataset using the same method is a well-known problem in this field (He and Yu, 2010). Nevertheless, our algorithm achieved a stable discriminative feature set through the combination of DO and Wilk’s lambda.

Our results were supported by the cancer-related terms provided by the DAVID tool in the enrichment analysis of both the colon and lung datasets. The analysis of colon cancer can be divided into five main aspects. First, uncontrolled bile secretion was identified as an environmental factor that promotes colon cancer progression (Raufman et al., 2015). Second, alterations in epithelial ion transport are a frequently observed problem in carcinogenesis (Davies et al., 1991). Third, bicarbonate administration has been shown to selectively reduce tumor aggressiveness by increasing pH (Robey and Martin, 2011). In addition, nitrogen metabolism is often disturbed in various cancers to promote cell survival (Kurmi and Haigis, 2020). Finally, pancreatitis (Ji et al., 2015) and pancreatic metastases (Bush et al., 2020) are rare side effects observed in colorectal cancer patients.

The enriched genes were observed to be biologically meaningful in colon and lung cancer cases. The enriched genes for colon cancer included *ATP1A2*, *SCNN1B*, *SLC10A2*, *SLC17A8*, *SLC4A4*, *ABCB11*, *SLC51B*, *SLC51A*, *SCN7A*, *SCN11A*, *FXYD1*, and *SCN9A*. With the exception of *ATP1A2*, the remaining genes were found to influence the development of colorectal cancer. Abnormal epithelial cell function has been reported to be responsible for 90% of all human cancers. *SCNN1B* is a gene that codes for the beta subunit of the epithelial sodium channel (ENaC). It has been observed that these channels control the behavior of malignant cancer cells (Liu et al., 2016). In the study by Qian et al. (2023), *SCNN1B* was observed to suppress the c-Raf and MAPK signaling cascade in colorectal cancer cell lines. Ectopic expression of *SCNN1B* in colorectal cancer cell lines resulted in the suppression of cell proliferation, induced apoptosis and cell cycle arrest, and suppressed cell migration. In addition to the cell line study, xenograft models were also used to investigate the tumor-suppressive function of the gene in animal models (Qian et al., 2023). Disruption of the enterohepatic bile acid cycle has been observed as a cause of intestinal disorders including cancer development (Xia et al., 2016). The apical sodium-dependent bile acid transporter (ASBT) is encoded by *SLC10A2*. In the study by Raufman et al., ASBT-deficient mice were compared with wild-type mice using azoxymethane (AOM)-induced tumor formation, and an increase in the size and number of colon tumors was observed in *SLC10A2*-silenced mice compared to the wild type (Raufman et al., 2015). Necroptosis is known as a programmed lytic cell death pathway observed in cells with deregulation based on inflammatory dysfunction (Najafov et al., 2017). Escape from necroptosis is known to play an important role in the growth of various tumor types including the colon (Yang et al., 2022). Solute carrier family member 4 (*SLCA4*) is one of the genes related to necroptosis and associated with poor progression in colorectal cancer patients. In the study by Yang et al. (2020), it was observed that lower expression of *SLCA4* caused poor prognosis in cancer patients with malignancies. ATP-binding cassette (ABC) transporters play a crucial role in the development of drug resistance due to the efflux of anticancer drugs from cancer cells. In the study by Hlavata et al. (2012), the efficacy of fluorouracil (5-FU)-containing treatment among colon cancer patients was investigated. The transcription levels of human ABCs were analyzed and patients with low *ABCB11* transcript levels had short disease-free intervals. Dysregulations in solute carrier proteins (SLCs) are known to cause the development of cancer due to the disruption of cellular metabolic homeostasis (Panda et al., 2020). The study by Lian et al. (2020) identified modules associated with colorectal cancer metastasis, and the results showed that 12 genes, including *SLC51B*, were correlated with two lncRNAs, RP11-396O20.2 and SNHG11, which are known to have stronger links to nodal sites. Voltage-gated sodium channels (NaVs) are known to be overexpressed in various cancers, including colorectal cancer, and are strongly associated with metastasis (Lopez-Charcas et al., 2023). In the study by Sun et al. (2019), a recurrent mutation of *SCN7A* was observed in brain metastasis tissues from metastatic patients. NaV1.7 is encoded by *SCN9A*. In the study by Xia et al. (2016), the expression of *SCN9A* correlated with the expression of the oncoprotein metastasis-associated in colon cancer-1 (MACC1), which significantly influences the development, invasion, and metastasis of various malignant cancers (Lv et al., 2023). The members of the FXYD gene family are small ion transport regulators that interact with Na+/K+-ATPase. It has been observed that these family members play important roles in the development of various types of cancer. In the study by Jin et al. (2021), *FXYD1* was associated with poor overall survival in colorectal cancer patients.

The lung cancer-enriched genes included *AQP4*, *HBA1*, *HBA2*, *HBB*, *HBM*, *GGTLC1*, *GGTLC2*, *GGTLC3*, *GPM6A*, *SFTPC*, *IL6*, *MYOC*, and *EPB42*. It is well known that the immune system plays a role in the development of lung cancer and the prognostic process. In the study by Zhu et al. (2023), the immune infiltration of LUAD was investigated. It was found that 12 hub genes, including *HBA2*, may be involved in LUAD progression via immune-related signaling pathways. Circulating tumor cells (CTCs) are known as cancer cells that detach from the solid tumor and enter the bloodstream. This group of cells contains a population of metastatic progenitors that are important for cancer progression (Castro Giner and Aceto, 2020). In the study by Zheng et al. (2017), a significant reduction in CTC-derived lung metastases was observed in *HBB*-negative CTC cultures. Aquaporins (AQPs) are channel-forming membrane proteins that have been reported to influence cancer cell growth, migration, invasion, and angiogenesis (Moon et al., 2022). In the study by Xie et al. (2012), AQP1 and AQP4 were analyzed for their influence on the invasive property of lung cancer cells. A significant reduction in the migration of AQP1 shRNA and AQP4 shRNA cells was observed compared to control lung cancer cells. Gamma-glutamyl transferase light chain 1 (*GGTLC1*) is one of the genes involved in glutamine biosynthesis. It has been observed that glutamine metabolism is increased in cancer cells and is associated with *Myc* downregulation related to the Warburg effect. The study by Kim et al. (2013) found that *GGTLC1* may be influenced by *NKX2-1*, an oncogene amplified in cases of NSCLC. According to that study, this situation was thought to result in cancer cells focusing on pathways required for rapid growth and metabolic requirements (Kim et al., 2013). Lymph node metastasis in lung cancer patients is an important factor in overall survival. The study by Dong et al. (2019) found significant differences in DEGs in patients with stage T1–2 and T3–4 disease. A top-ten DEG list was created for each stage, comparing metastatic and nonmetastatic cases. In both, upregulation of the *MYOC* gene was observed, indicating potential efficacy in triggering metastasis (Dong et al., 2019). miR-629-3p is a major miRNA that is upregulated, especially in cases of human breast cancer, and affects cell viability and migration. In the study by Li et al. (2019), it was observed that miR-629-3P-mediated downregulation of *SFTPC* promoted tumor proliferation and invasion of lung cancer cells. In addition, downregulation of *SFPTC* was observed in patients with poor survival rates. The association between type 2 diabetes and a high risk of developing cancer has also been reported (Travier et al., 2007). The glycosylated form of HbA1 (HbA1c), which provides an estimate of a person’s blood glucose level in the last 3 months, has implications for diabetes (Nitin, 2010). In the study by Travier et al. (2007), it was found that an increase in HbA1c levels poses a risk for respiratory cancers. Cytokines are small proteins that play important roles in cancer development (Abolfathi et al., 2021). IL6, a pleiotropic cytokine, functions in the regulation of the immune system (Yao et al., 2014). In the study by Liu et al. (2020), IL6 was observed as a critical element for NSCLC as it affects the epithelial-to-mesenchymal transition and metastasis and causes drug resistance. GPM6A is a neuronal membrane glycoprotein that has been detected in various cancers such as those of the colon, liver, and lungs. In the study by Zhang et al. (2022), it was observed that induced overexpression of GPM6A in a mouse model of lung cancer delayed and reduced tumor growth.

Due to the long time and high costs associated with drug discovery in the field of cancer biology, drug repurposing is becoming an increasingly attractive and promising solution (Issa et al., 2020). Focusing on using existing disease-related genes can be advantageous for our algorithm and beneficial for the area of drug repurposing (Antolin et al., 2016). Our algorithm has the potential to facilitate the development of new treatment procedures that require fewer drugs, resulting in fewer cumulative effects on patients.

The present version of the algorithm can successfully classify tumor/normal states through the use of RNA-Seq expression datasets. Initially, we attempted to use count data for the analysis, which unfortunately failed to discriminate between the two states. As a result, we changed our approach to focus on normalized expression datasets. We found that the normalized forms of FPKM and RPKM were adequate for achieving accurate sample classification. In PCA analysis, both RNA-Seq and microarray datasets were used to test the diagnostic performance of the acquired gene sets. The tests using RNA-Seq involved both count and normalized datasets. In the case of microarrays, only normalized datasets from the Affymetrix platform were used in the facilitated analysis.

The generated gene sets showed a moderate prognostic effect, and we were able to achieve optimal separation of tumor/normal states in various datasets using our gene sets despite the use of different platforms and normalization methods. Furthermore, our approach demonstrated strong predictive performance, as evidenced by high AUC values that were independent of input size. In addition, our algorithm performed well in terms of classification when compared to other SVM-based and clustering approaches that used the same datasets.

This study demonstrated the effectiveness of using disease-associated genes and Wilk’s lambda criterion to construct an SVM classification model for detecting cancer biomarkers. We anticipate that our approach will prove useful for further analyses and yield comparable results in the field of cancer research. An R package of our algorithm in the form of a GUI is available in Bioconductor (http://doi.org/10.18129/B9.bioc.SVMDO) and GitHub (https://github.com/robogeno/SVMDO).

## Supplementary Data

**Table S1:** Cancer-associated disease terms of combined discriminative gene sets.

Dataset	Disease Ontology ID	Disease Ontology Term
**COAD**	DOID:0060108	brain glioma
	DOID:1319	brain cancer
	DOID:3620	central nervous system cancer
	DOID:3829	pituitary adenoma
	DOID:60009	pituitary gland benign neoplasm
	DOID:657	adenoma
	DOID:0060084	cell type benign neoplasm
	DOID:2226	myeloproliferative neoplasm
	DOID:4960	bone marrow cancer
	DOID:0070004	myeloid neoplasm
	DOID:3500	gallbladder adenocarcinoma
	DOID:2021	placenta cancer
	DOID:3594	choriocarcinoma
	DOID:707	B-cell lymphoma
	DOID:4948	gallbladder carcinoma
	DOID:8552	chronic myeloid leukemia
	DOID:3121	gallbladder cancer
	DOID:0060060	non-Hodgkin lymphoma
	DOID:9952	acule lymphoblastic leukemia
	DOID:0060058	lymphoma
	DOID:9119	acute myeloid leukemia
	DOID:12603	acute leukemia
	DOID:4607	biliary tract cancer
	DOID:8692	myeloid leukemia
	DOID:9538	multiple myeloma
	DOID:4450	renal cell carcinoma
	DOID:3459	breast carcinoma
	DOID:5940	malignant peripheral nerve sheath tumor
	DOID:3193	peripheral nerve sheath neoplasm
	DOID:3192	neurilemmoma
	DOID:2001	neuroma
	DOID:0060115	nervous system benign neoplasm
	DOID:1192	peripheral nervous system neoplasm
	DOID:0060085	organ system benign neoplasm
	DOID:5389	oxyphilic adenoma
	DOID:0060089	endocrine organ benign neoplasm
	DOID:2151	malignant ovarian surface epithelial-stromal neoplasm
	DOID:4001	ovarian carcinoma
	DOID:2152	ovary epithelial cancer
	DOID:2394	ovarian cancer
	DOID:1115	sarcoma
	DOID:2621	autonomic nervous system neoplasm
	DOID:769	neuroblastoma
	DOID:4766	embryoma
	DOID:688	embryonal cancer
	DOID:2994	germ cell cancer
	DOID:0060121	integumentary system benign neoplasm
	DOID:3165	skin benign neoplasm
	DOID:3113	papillary carcinoma
	DOID:4007	bladder carcinoma
	DOID:5520	head and neck squamous cell carcinoma
	DOID:11054	urinary bladder cancer
	DOID:1542	head and neck carcinoma
	DOID:11934	head and neck cancer
	DOID:3910	lung adenocarcinoma
	DOID:3962	thyroid gland follicular carcinoma
	DOID:3969	thyroid gland papillary carcinoma
	DOID:0080524	thyroid gland adenocarcinoma
	DOID:0080525	differentiated thyroid gland carcinoma
	DOID:3963	thyroid gland carcinoma
	DOID:1781	thyroid gland cancer
	DOID:0050771	pheochromocytoma
	DOID:3498	pancreatic ductal adenocarcinoma
	DOID:4074	pancreatic adenocarcinoma
	DOID:4905	pancreatic carcinoma
	DOID:3713	ovary adenocarcinoma
	DOID:0080364	malignant adenoma
	DOID:3111	cystadenocarcinoma
	DOID:3114	serous cystadenocarcinoma
	DOID:60004	malignant cystadenoma
	DOID:3355	fibrosarcoma
	DOID:201	connective tissue cancer
	DOID:0060100	musculoskeletal system cancer
	DOID:4914	esophagus adenocarcinoma
	DOID:127	leiomyoma
	DOID:8719	in situ carcinoma
	DOID:0060071	pre-malignant neoplasm
	DOID:1107	esophageal carcinoma
	DOID:1036	chronic leukemia
	DOID:1040	chronic lymphocytic leukemia
	DOID:5041	esophageal cancer
	DOID:10534	stomach cancer
	DOID:4896	bile duct adenocarcinoma
	DOID:4947	cholangiocarcinoma
	DOID:4897	bile duct carcinoma
	DOID:4606	bile duct cancer
	DOID:3347	osteosarcoma
	DOID:0080639	bone sarcoma
	DOID:184	bone cancer
	DOID:2871	endometrial carcinoma
	DOID:1380	endometrial cancer
	DOID:363	uterine cancer
	DOID:3748	esophagus squamous cell carcinoma
	DOID:2645	benign mesothelioma
	DOID:1294	vulva carcinoma
	DOID:1245	vulva cancer
	DOID:4467	clear cell renal cell carcinoma
	DOID:0060095	uterine benign neoplasm
	DOID:0060086	female reproductive organ benign neoplasm
	DOID:0050622	reproductive organ benign neoplasm
	DOID:3565	meningioma
	DOID:2671	transitional cell carcinoma
	DOID:3744	cervical squamous cell carcinoma
	DOID:2893	cervix carcinoma
	DOID:3069	malignant astrocytoma
	DOID:4362	cervical cancer
	DOID:3070	high grade glioma
	DOID:768	retinoblastoma
	DOID:771	retinal cell cancer
	DOID:4645	retinal cancer
	DOID:0060116	sensory system cancer
	DOID:2174	ocular cancer
	DOID:962	neurofibroma
	DOID:452	pleomorphic adenoma
	DOID:1790	malignant mesothelioma
	DOID:0050624	gastrointestinal system benign neoplasm
	DOID:0060122	integumentary system cancer
	DOID:4159	skin cancer
	DOID:9261	nasopharynx carcinoma
	DOID:0060119	pharynx cancer
	DOID:5517	stomach carcinoma
	DOID:0060082	breast benign neoplasm
	DOID:0060097	thoracic benign neoplasm
	DOID:0050860	colorectal adenoma
	DOID:4610	intestinal benign neoplasm
	DOID:0080199	colorectal carcinoma
	DOID:3717	gastric adenocarcinoma
**LUSC**	DOID:3493	signet ring cell adenocarcinoma
	DOID:299	adenocarcinoma
	DOID:2621	autonomic nervous system neoplasm
	DOID:769	neuroblastoma
	DOID:1192	peripheral nervous system neoplasm
	DOID:4450	renal cell carcinoma
	DOID:4467	clear cell renal cell carcinoma
	DOID:3008	invasive ductal carcinoma
	DOID:3247	rhabdomyosarcoma
	DOID:3007	breast ductal carcinoma
	DOID:4043	skeletal muscle cancer
	DOID:0080199	colorectal carcinoma
	DOID:4045	muscle cancer
	DOID:3910	lung adenocarcinoma
	DOID:3459	breast carcinoma
	DOID:962	neurofibroma
	DOID:452	pleomorphic adenoma
	DOID:3193	peripheral nerve sheath neoplasm
	DOID:1790	malignant mesothelioma
	DOID:0050624	gastrointestinal system benign neoplasm
	DOID:657	adenoma
	DOID:0060085	organ system benign neoplasm
	DOID:0060084	cell type benign neoplasm
	DOID:3683	lung benign neoplasm
	DOID:255	hemangioma
	DOID:0060091	cardiovascular organ benign neoplasm
	DOID:0050621	respiratory system benign neoplasm
	DOID:11054	urinary bladder cancer
	DOID:9952	acute lymphoblastic leukemia
	DOID:9119	acute myeloid leukemia
	DOID:12603	acute leukemia
	DOID:8692	myeloid leukemia
	DOID:3962	thyroid gland follicular carcinoma
	DOID:4948	gallbladder carcinoma
	DOID:3121	gallbladder cancer
	DOID:3969	thyroid gland papillary carcinoma
	DOID:0080524	thyroid gland adenocarcinoma
	DOID:0080525	differentiated thyroid gland carcinoma
	DOID:5683	hereditary breast ovarian cancer syndrome
	DOID:3963	thyroid gland carcinoma
	DOID:1781	thyroid gland cancer
	DOID:4607	biliary tract cancer
	DOID:2151	malignant ovarian surface epithelial-stromal neoplasm
	DOID:4001	ovarian carcinoma
	DOID:2152	ovary epithelial cancer
	DOID:2394	ovarian cancer
	DOID:0060318	acute promyelocytic leukemia
	DOID:3304	germinoma
	DOID:0080830	childhood low-grade glioma
	DOID:4851	pilocytic astrocytoma
	DOID:171	neuroectodermal tumor
	DOID:3275	thymoma
	DOID:0080829	low grade glioma
	DOID:3277	thymus cancer
	DOID:0060083	immune system cancer
	DOID:3069	malignant astrocytoma
	DOID:3070	high grade glioma
	DOID:4766	embryoma
	DOID:688	embryonal cancer
	DOID:2994	germ cell cancer
	DOID:4914	esophagus adenocarcinoma
	DOID:3907	lung squamous cell carcinoma
	DOID:2871	endometrial carcinoma
	DOID:1380	endometrial cancer
	DOID:1107	esophageal carcinoma
	DOID:363	uterine cancer
	DOID:5041	esophageal cancer
	DOID:10286	prostate carcinoma
	DOID:1115	sarcoma
	DOID:1542	head and neck carcinoma
	DOID:11934	head and neck cancer
	DOID:1520	colon carcinoma
	DOID:219	colon cancer
	DOID:3587	pancreatic ductal carcinoma
	DOID:4905	pancreatic carcinoma
	DOID:0050865	tongue squamous cell carcinoma
	DOID:5520	head and neck squamous cell carcinoma
	DOID:8567	Hodgkin’s lymphoma
	DOID:0060058	lymphoma
	DOID:0060071	pre-malignant neoplasm
	DOID:3347	osteosarcoma
	DOID:0080639	bone sarcoma
	DOID:184	bone cancer
	DOID:201	connective tissue cancer
	DOID:0060108	brain glioma
	DOID:1319	brain cancer
	DOID:3620	central nervous system cancer
	DOID:1036	chronic leukemia
	DOID:1040	chronic lymphocytic leukemia
	DOID:175	vascular cancer
	DOID:176	cardiovascular cancer
	DOID:3498	pancreatic ductal adenocarcinoma
	DOID:3068	glioblastoma
	DOID:4074	pancreatic adenocarcinoma
	DOID:3376	bone osteosarcoma
	DOID:1800	neuroendocrine carcinoma
	DOID:0050938	breast lobular carcinoma
	DOID:3457	invasive lobular carcinoma
	DOID:3308	embryonal carcinoma
	DOID:4440	seminoma
	DOID:4896	bile duct adenocarcinoma
	DOID:4947	cholangiocarcinoma
	DOID:4897	bile duct carcinoma
	DOID:4606	bile duct cancer
	DOID:4418	cutaneous fibrous histiocytoma
	DOID:4415	fibrous histiocytoma
	DOID:4231	histiocytoma
	DOID:0060123	connective tissue benign neoplasm
	DOID:0060099	musculoskeletal system benign neoplasm
	DOID:3717	gastric adenocarcinoma
	DOID:5517	stomach carcinoma
	DOID:10534	stomach cancer
	DOID:3479	uveal cancer
	DOID:6039	uveal melanoma
	DOID:3713	ovary adenocarcinoma
	DOID:5183	hereditary Wilms’ tumor
	DOID:707	B-cell lymphoma
	DOID:8552	chronic myeloid leukemia
	DOID:2154	nephroblastoma
	DOID:0060060	non-Hodgkin lymphoma
	DOID:0060116	sensory system cancer
	DOID:2174	ocular cancer
	DOID:9538	multiple myeloma
	DOID:0070004	myeloid neoplasm
	DOID:4960	bone marrow cancer
	DOID:3744	cervical squamous cell carcinoma
	DOID:0060122	integumentary system cancer
	DOID:4159	skin cancer
	DOID:2893	cervix carcinoma
	DOID:4362	cervical cancer
	DOID:0050860	colorectal adenoma
	DOID:4610	intestinal benign neoplasm
	DOID:5409	lung small cell carcinoma
**LUAD**	DOID:0050685	small cell carcinoma
	DOID:6536	plasma cell neoplasm
	DOID:1785	pituitary cancer
	DOID:656	adrenal adenoma
	DOID:0050523	adult T-cell leukemia/lymphoma
	DOID:0050625	biliary tract benign neoplasm
	DOID:0060090	central nervous system benign neoplasm
	DOID:8632	Kaposi’s sarcoma
	DOID:5395	functioning pituitary adenoma
	DOID:0060121	integumentary system benign neoplasm
	DOID:235	colonic benign neoplasm
	DOID:3165	skin benign neoplasm
	DOID:169	neuroendocrine tumor
	DOID:1993	rectum cancer
	DOID:706	mature B-cell neoplasm
	DOID:5603	T-cell acute lymphoblastic leukemia
	DOID:3355	fibrosarcoma
	DOID:3117	hepatobiliary benign neoplasm
	DOID:2513	basal cell carcinoma
	DOID:2600	laryngeal carcinoma
	DOID:255	hemangioma
	DOID:0060091	cardiovascular organ benign neoplasm
	DOID:0060095	uterine benign neoplasm
	DOID:2596	larynx cancer
	DOID:8567	Hodgkin’s lymphoma
	DOID:0060086	female reproductive organ benign neoplasm
	DOID:0050622	reproductive organ benign neoplasm
	DOID:0060115	nervous system benign neoplasm
	DOID:4610	intestinal benign neoplasm
	DOID:707	B-cell lymphoma
	DOID:3829	pituitary adenoma
	DOID:60009	pituitary gland benign neoplasm
	DOID:3371	chondrosarcoma
	DOID:0060089	endocrine organ benign neoplasm
	DOID:0050866	oral squamous cell carcinoma
	DOID:3451	skin carcinoma
	DOID:9261	nasopharynx carcinoma
	DOID:3744	cervical squamous cell carcinoma
	DOID:0060119	pharynx cancer
	DOID:0080199	colorectal carcinoma
	DOID:0060060	non-Hodgkin lymphoma
	DOID:3717	gastric adenocarcinoma
	DOID:127	leiomyoma
	DOID:3498	pancreatic ductal adenocarcinoma
	DOID:2871	endometrial carcinoma
	DOID:8618	oral cavity cancer
	DOID:0060122	integumentary system cancer
	DOID:4159	skin cancer
	DOID:2893	cervix carcinoma
	DOID:9952	acute lymphoblastic leukemia
	DOID:0060058	lymphoma
	DOID:3069	malignant astrocytoma
	DOID:4362	cervical cancer
	DOID:4896	bile duct adenocarcinoma
	DOID:4947	cholangiocarcinoma
	DOID:4074	pancreatic adenocarcinoma
	DOID:5517	stomach carcinoma
	DOID:1115	sarcoma
	DOID:4897	bile duct carcinoma
	DOID:5520	head and neck squamous cell carcinoma
	DOID:4606	bile duct cancer
	DOID:1380	endometrial cancer
	DOID:9119	acute myeloid leukemia
	DOID:363	uterine cancer
	DOID:1036	chronic leukemia
	DOID:1040	chronic lymphocytic leukemia
	DOID:12603	acute leukemia
	DOID:3910	lung adenocarcinoma
	DOID:1542	head and neck carcinoma
	DOID:4607	biliary tract cancer
	DOID:0080639	bone sarcoma
	DOID:11934	head and neck cancer
	DOID:8692	myeloid leukemia
	DOID:184	bone cancer
	DOID:9538	multiple myeloma
	DOID:4905	pancreatic carcinoma
	DOID:2151	malignant ovarian surface epithelial-stromal neoplasm
	DOID:4001	ovarian carcinoma
	DOID:2152	ovary epithelial cancer
	DOID:657	adenoma
	DOID:3070	high grade glioma
	DOID:4766	embryoma
	DOID:0070004	myeloid neoplasm
	DOID:688	embryonal cancer
	DOID:2621	autonomic nervous system neoplasm
	DOID:769	neuroblastoma
	DOID:201	connective tissue cancer
	DOID:4960	bone marrow cancer
	DOID:1192	peripheral nervous system neoplasm
	DOID:10534	stomach cancer
	DOID:0060085	organ system benign neoplasm
	DOID:2994	germ cell cancer
	DOID:2394	ovarian cancer
	DOID:0060100	musculoskeletal system cancer
	DOID:4450	renal cell carcinoma
	DOID:0060084	cell type benign neoplasm
	DOID:3459	breast carcinoma
	DOID:4682	extrahepatic bile duct carcinoma
	DOID:3493	signet ring cell adenocarcinoma
	DOID:5389	oxyphilic adenoma
	DOID:12689	acoustic neuroma
	DOID:2876	laryngeal squamous cell carcinoma
	DOID:8029	sporadic breast cancer
	DOID:3192	neurilemmoma
	DOID:2001	neuroma
	DOID:3376	bone osteosarcoma
	DOID:8791	breast carcinoma in situ
	DOID:3008	invasive ductal carcinoma
	DOID:3458	breast adenocarcinoma
	DOID:0060099	musculoskeletal system benign neoplasm
	DOID:4007	bladder carcinoma
	DOID:234	colon adenocarcinoma
	DOID:3565	meningioma
	DOID:2671	transitional cell carcinoma
	DOID:3007	breast ductal carcinoma
	DOID:5409	lung small cell carcinoma
	DOID:8719	in situ carcinoma
	DOID:3748	esophagus squamous cell carcinoma
	DOID:299	adenocarcinoma
	DOID:0060071	pre-malignant neoplasm
	DOID:10286	prostate carcinoma
	DOID:3620	central nervous system cancer
	DOID:1520	colon carcinoma
	DOID:1107	esophageal carcinoma
	DOID:3347	osteosarcoma
	DOID:11054	urinary bladder cancer
	DOID:5041	esophageal cancer
	DOID:219	colon cancer
	DOID:5683	hereditary breast ovarian cancer syndrome
	DOID:3308	embryonal carcinoma
	DOID:4440	seminoma
	DOID:4914	esophagus adenocarcinoma
	DOID:3907	lung squamous cell carcinoma
	DOID:3702	cervical adenocarcinoma
	DOID:0060074	ductal carcinoma in situ
	DOID:4928	intrahepatic cholangiocarcinoma
	DOID:2226	myeloproliferative neoplasm
	DOID:8552	chronic myeloid leukemia
	DOID:3068	glioblastoma
	DOID:5183	hereditary Wilms’ tumor
	DOID:2154	nephroblastoma
	DOID:1800	neuroendocrine carcinoma
	DOID:5157	benign pleural mesothelioma
	DOID:1790	malignant mesothelioma
	DOID:5158	pleural cancer
	DOID:7474	malignant pleural mesothelioma
	DOID:0050621	respiratory system benign neoplasm
	DOID:3969	thyroid gland papillary carcinoma
	DOID:0080524	thyroid gland adenocarcinoma
	DOID:0080525	differentiated thyroid gland carcinoma
	DOID:3963	thyroid gland carcinoma
	DOID:1781	thyroid gland cancer
	DOID:4051	alveolar rhabdomyosarcoma
	DOID:3246	embryonal rhabdomyosarcoma
	DOID:3247	rhabdomyosarcoma
	DOID:4043	skeletal muscle cancer
	DOID:4045	muscle cancer

## Figures and Tables

**Table 1:** Transcriptome datasets employed in the present study.

Tumor	Dataset ID	Sample Size		Platform	Normalization procedure
		Normal	Tumor		
COAD	TCGA	41	478	RNA-Seq	FPKM
	GSE4107	10	12	Microarray	MAS5, scaled to 500
	GSE8671	32	32	Microarray	MAS5
	GSE24514	15	34	Microarray	RMA
	GSE32323	17	17	Microarray	RMA
	GSE95132	10	10	RNA-Seq	Count (Not Normalized)
LUSC	TCGA	49	502	RNA-Seq	FPKM
	GSE84784	9	9	Microarray	RMA
LUAD	GSE2514	19	20	Microarray	MAS5
	GSE40419	77	87	RNA-Seq	RPKM
	GSE148036	5	5	RNA-Seq	Count (Not Normalized)

**Table 2:** Diagnostic performance of gene sets as a result of principal component analysis (mean ± SEM).

Input Size	Accuracy (Microarray)	Sensitivity (Microarray)	Specificity (Microarray)	Accuracy (RNA-Seq)	Sensitivity (RNA-Seq)	Specificity (RNA-Seq)
50	0.92 ± 0.02	0.93 ± 0.073	0.92 ± 0.03	0.93 ± 0.06	0.97 ± 0.02	0.85 ± 0.10
100	0.95 ± 0.02	0.93 ± 0.03	0.97 ± 0.01	0.95 ± 0.04	0.94 ± 0.03	0.93 ± 0.05
200	0.94 ± 0.02	0.91 ± 0.04	0.98 ± 0.01	0.97 ± 0.03	0.97 ± 0.02	0.95 ± 0.03
300	0.95 ± 0.02	0.92 ± 0.03	0.98 ± 0.01	0.97 ± 0.02	0.99 ± 0.01	0.95 ± 0.03
400	0.96 ± 0.05	0.94 ± 0.04	0.98 ± 0.01	0.97 ± 0.02	0.98 ± 0.01	0.95 ± 0.03
500	0.95 ± 0.02	0.91 ± 0.05	0.99 ± 0.01	0.97 ± 0.02	0.98 ± 0.01	0.95 ± 0.03

**Table 3:** Lists of prognostic genes in TCGA-COAD and TCGA-LUSC datasets at different input sizes.

Dataset	n=50	n=100	n=200	n=300	n=400	n=500
TCGA-COAD	*GUCA2B*	*CLCA4*	*CHP2*	*CA4*	*CA4*	*CA4*
		*SLC30A10*	*CA4*	*CDKN2B-AS1*	*CDKN2B-AS1*	*CDKN2B-AS1*
		*CDKN2B-AS1*	*CDKN2B-AS1*	*CHP2*	*CHGA*	*CLCA4*
		*CA4*	*SLC30A10*	*UGT2B17*	*SLC30A10*	*CHP2*
		*GUCA2B*	*CLCA4*	*CLCA4*	*GALR1*	*UGT2B17*
		*CD177*	*UGT2B17*	*VEGFD*	*VEGFD*	*HBE1*
			*VEGFD*	*ALPI*	*CLCA4*	*ADAMDEC1*
			*HBE1*	*SLC30A10*	*UGT2B17*	
			*CHGA*	*CD177*	*ALPI*	
			*CD177*	*SFRP5*	*CHP2*	
					*CD177*	
TCGA-LUSC	*AGER*	*ADH1B*	*GPIHBP1*	*FHL5*	*MRC1*	*FHL5*
	*SFTPC*	*TNXB*	*AQP4*	*MARCO*	*ZBTB16*	*CLIC5*
	*GPIHBP1*	*CLEC4M*	*GGTLC1*	*GPIHBP1*	*FHL5*	*MRC1*
	*PRG4*	*GP9*	*ANKRD1*	*ZBTB16*	*GPIHBP1*	*GPIHBP1*
	*CLIC5*	*SFTPC*	*CLIC5*	*TNNC1*	*ADH1B*	*ZBTB16*
		*SFTPA1*	*ZBTB16*	*GGTLC1*	*AQP4*	*C7*
			*AGER*	*TNXB*	*MARCO*	*OGN*
			*PRG4*	*SFTPA1*	*CLIC5*	*MARCO*
			*TNXB*	*AQP4*	*COL4A3*	*ABCA3*
			*SFTPA1*	*SFTPA2*	*TNNC1*	*GGTLC2*
			*TNNC1*	*CLIC5*	*TNXB*	*COL4A3*
			*GP9*	*TCF21*	*SFTPA1*	*CPB2*
			*SFTPA2*		*SFTPC*	*DLC1*
			*PRG4*		*ASPA*	*SOX17*
			*OGN*		*GP9*	*SFTPA2*
			*FHL5*		*ABCA3*	*LRRK2*
						*AGTR2*
						*ASPA*

**Table 4:** Prognostic performances of genes in TCGA-COAD and TCGA-LUSC datasets.

Dataset	Genes	Log-Rank p value	Hazard Ratio
TCGA-COAD	*GALR1*	0.013	0.61
	*SFRP5*	0.037	0.66
	*ADAMDEC1*	0.034	1.53
	*CHP2*	0.040	1.51
	*UGT2B17*	0.008	1.70
	*VEGFD*	0.040	0.66
	*ALPI*	0.016	1.62
	*CHGA*	0.015	1.64
	*CDKN2B-AS1*	0.013	1.65
	*CA4*	0.034	1.54
	*CLCA4*	0.001	1.92
	*CD177*	0.047	1.50
	*SLC30A10*	0.017	1.62
	*GUCA2B*	0.0002	2.07
	*HBE1*	0.011	1.67
TCGA-LUSC	*OLR1*	0.032	0.75
	*C7*	0.027	1.35
	*SFTPB*	0.011	1.41
	*AGTR2*	0.035	1.33
	*SOX17*	0.038	1.32
	*LRRK2*	0.009	1.42
	*ABCA3*	0.007	1.43
	*COL4A3*	0.034	1.33
	*MRC1*	0.022	1.36
	*ASPA*	0.004	1.47
	*DLC1*	0.025	1.35
	*MARCO*	0.002	1.52
	*FHL5*	0.005	1.45
	*OGN*	0.033	1.33
	*GGTLC2*	0.031	1.34
	*ZBTB16*	0.001	1.54
	*AQP4*	0.048	0.76
	*SFTPA2*	0.048	1.31
	*TCF21*	0.016	1.38
	*SFTPA1*	0.010	1.42
	*TNXB*	0.006	1.44
	*CLEC4M*	0.005	1.45
	*GP9*	0.016	1.38
	*TNNC1*	0.024	1.36
	*ADH1B*	0.021	1.36
	*CPB2*	0.007	1.43
	*GPIHBP1*	0.004	1.47
	*CLIC5*	0.049	1.31
	*PRG4*	0.022	1.36
	*GGTLC1*	0.009	1.42
	*ANKRD1*	0.036	1.33
	*AGER*	0.002	1.52
	*SFTPC*	0.040	1.32

**Table 5:** Alternative methods employed in performance comparisons.

Method	Reference study
Proposed Multi-objective Adaptive Binary Particle Swarm Optimization (AMOPSO)	(Shahbeig et al., 2018)
Multi-objective Binary Genetic Algorithm (MOBGA)	
Multi-objective traditional Binary Particle Swarm Optimization (MOBPSO)	
Multi-objective Binary Teaching Learning Based Optimization (MOBTLBO)	
Single-objective Binary Genetic Algorithm (SOBGA)	
Single-objective traditional Binary Particle Swarm Optimization (SOBPSO)	
Single-objective Binary Teaching Learning Based Optimization (SOBTLBO)	
Graph Regularized Nonnegative Matrix Factorization (GNMF)	(Wang et al., 2019)
K-means Clustering (KMC)	
Proposed Laplacian regularized Low-Rank Representation (LLRR)	
Low-Rank Representation (LRR)	
Mixed-norm Laplacian Regularized Low-Rank Representation (MLLRR)	
Penalized Matrix Decomposition (PMD)	
Segmented Principal Component Analysis (SPCA)	

## Data Availability

The RNA-Seq and clinical datasets used in testing the SVM-DO algorithm can be downloaded from https://github.com/robogeno/svmdo_datasets.
